# Stereotactic Body Radiotherapy Is Effective in Modifying the Tumor Genome and Tumor Immune Microenvironment in Non-Small Cell Lung Cancer or Lung Metastatic Carcinoma

**DOI:** 10.3389/fimmu.2020.594212

**Published:** 2021-01-22

**Authors:** Pu Zhou, Diangang Chen, Bo Zhu, Wei Chen, Qichao Xie, Yali Wang, Qiulin Tan, Bibo Yuan, Xuejiao Zuo, Changlin Huang, Hongfan Zhu, Guanghui Li

**Affiliations:** ^1^Institute of Cancer, Xinqiao Hospital, Army Medical University, Chongqing, China; ^2^Zhongyuan Union Clinical Laboratory Co. Ltd, Beijing, China; ^3^Department of Oncology, Third Affiliated Hospital, Chongqing Medical University, Chongqing, China; ^4^Department of Pathology, Xinqiao Hospital, Army Medical University, Chongqing, China; ^5^Department of Internal Medicine, Army 956 Hospital, Linzhi, China

**Keywords:** stereotactic body radiotherapy, tumor microenvironment, tumor-infiltrating immune cells, T-cell receptor, whole-exome sequencing, RNA sequencing

## Abstract

**Background and Purpose:**

To directly reveal the change in genome mutation, RNA transcript of tumor cells, and tumor microenvironment (TME) after stereotactic body radiotherapy (SBRT) in paired human lung tumor specimens.

**Materials and Methods:**

Paired tumor samples were collected from 10 patients with non-small cell lung cancer (NSCLC) or lung metastatic carcinoma within a week before and after SBRT. DNA and RNA of tumor tissues was extracted from the paired samples. Whole-exome and RNA sequencing assays were performed by next-generation sequencing. Gene mutation, genomic expression, T-cell receptor (TCR) repertoire, and profiling of tumor-infiltrating immune cells were analyzed through bioinformatics analysis in paired tumor samples. CD8+ T-cell infiltration and PD-L1 expressions were detected by immunostaining in tumor tissues.

**Results:**

The diversity of TCR repertoire and PD-L1 expression increased significantly in the TME, and the most enriched term of the gene ontology analysis was the immune response gene after receiving SBRT. SBRT induced neo-mutation of genes in tumor cells but did not increase tumor mutation burden in tumor tissues. TME displayed complex immune cell changes and infiltration and expression of immune-regulating factors such as C-X-C motif chemokine (CXCL) 10, CXCL16, interferons (IFNs), and IFN receptors. CD8+ T-cells in tumor tissues did not improve significantly after SBRT while the infiltrating TH1 and TH2 cells decreased remarkably.

**Conclusion:**

SBRT improved the TCR repertoire diversity and PD-L1 expression in the TME and induced neo-mutation of genes in tumor cells but did not increase CD8+ T-cell infiltration and IFN expression in the tumor tissue within a week.

## Introduction

Significant synergy between radiotherapy and treatment with immune checkpoint inhibitors (ICIs) would potentiate the effect of irradiation on antitumor immune response. Immunotherapy combined with radiotherapy is more effective than monotherapy alone for various types of tumors (1–3). Radiotherapy could even rescue a nivolumab-refractory immune response in patients with metastatic lung carcinoma (4). Radiation-induced antitumor immune response in patients varied widely depending on the doses delivered, fraction, and irradiation site of radiotherapy (5). Several reports confirmed that stereotactic body radiotherapy (SBRT) is more potent in terms of improving ICI anti-tumor response compared with conventional fraction radiation in patients with non-small cell lung cancer (NSCLC) (6, 7).

Although numerous reports demonstrated that radiotherapy enhanced the therapeutic effect of immunotherapy (8), the direct impact of SBRT on the tumor cell genome and the tumor microenvironment (TME) remains unclear in patients with cancer. Here, we examined the genomic variance of cancer cells, immune cell infiltration, and immune-regulating factor expression of the TME in 10 patients with lung tumors who received SBRT.

## Materials and Methods

### Study Patients

Thirteen patients aged 43–68 years were included. They had histologically documented malignant tumor, recurrent or metastatic; World Health Organization (WHO) performance status score of 0 to 2; life expectancy of at least 3 months at day 1; and indication for local radiotherapy. Four patients had metastatic lung tumor from colorectal carcinoma (CRC), seven had NSCLC, one had metastatic esophageal carcinoma in the lungs, and one had metastatic cervical cancer in the lungs. The medical ethics committee of Xinqiao Hospital of Army Medical University approved the study (Ethical No: 2019-Res 032-1). All patients provided informed consent.

### Biopsy and Radiotherapy

Computed tomography-guided puncture biopsy of the lung deposit was performed a week before SBRT. Peripheral blood samples were collected in ethylenediaminetetraacetic acid vacutainer tubes at the same time. Mononuclear cells separated by centrifugation at 1,600 *g* were transferred to a new microcentrifuge tube and were fixed with a preserving buffer (Geneplus Biotech, China) for 24 h at 4°C and were then placed in the refrigerator (−80°C) to preserve sample integrity. Lung targeted tumors received 60 Gy irradiation in 10 fractions using Varian Triology Linear Accelerator (Varian Medical System Inc, USA). The second samples of irradiated tumors and peripheral white blood cells were collected within a week after radiotherapy in the same manner as the first collection. Three pieces of tissue were taken from each tumor biopsy. One piece was fixed with formalin for pathological examination and was set for quality control (QC) to ensure samples have adequate tumor cells. Two pieces were fixed with a preserving buffer (Geneplus Biotech, China) for 24 h at 4°C and were then placed in a refrigerator (−80°C) for RNA extraction and DNA extraction, respectively.

### Nucleic Acid Extraction, Library Construction, and Sequencing

Paired tumor samples and lymphocytes were submitted for next-generation sequencing (NGS) in Zhongyuan-Vcan Genetic Technology Co., Ltd (Tianjing, China). Genomic DNA was extracted from tumor tissues and blood cells using the TIANamp Genomic DNA Kit (Tiangen) for whole-exome sequencing (WES). Paired analysis of tumor and matched-lymphocyte samples was performed for definitive identification of somatic variations. DNA integrity was assessed by agarose gel electrophoresis. DNA concentrations were measured using a Qubit fluorometer (Thermo Fisher Scientific). WES libraries were constructed using the KAPA HyperPrep Kits (KAPA) and enriched using Agilent SureSelect^XT^ target enrichment kit ILM (Agilent). Genomic DNA from tumor paired samples and matched-lymphocyte samples were sequenced using the NextSeq 500 platform. Exome capture was performed using SureSelect^XT^ Human All Exon 60 MB (Agilent). Tumor exomes were sequenced at 200× coverage, while blood cell exomes were sequenced at 100×. Burrows-Wheeler Aligner (BWA) (version 0.7.15-r1140) was used to align sequences over the human reference genome HG19.

Total RNA was extracted from tumor tissues using TRIzol™ Reagent (Life Technologies) for transcriptome analyses. RNA integrity, purity, and concentration were evaluated using an Agilent 2100 Bioanalyzer (Agilent Technologies). Nanodrop and Qubit 3.0. RNA-seq libraries were constructed using KAPA mRNA HyperPrep Kit. WES and RNA-seq were performed in the Illumina Novaseq System. All libraries were sequenced on the Novaseq using 2×150 bp paired-end sequencing. Adaptor sequences were trimmed, and reads were processed into clean reads. Each RNA-seq data required approxiimately 10 G.

### Analysis of Gene Expression, Fusion Gene, Tumor-Infiltrating Immune Cells, and TCR Repertoire

The paired-end clean reads were mapped to the human reference genome GRCh37.75 using STAR (version 020201) with default parameters (9). A novel network flow algorithm used in StringTie (version 2.2.1) (10) was applied to perform transcript assembly and quantify gene expression in fragments per kilobase of transcript per million fragment mapped units for RNA-seq. Then, DESeq (version 1.24.0) (11) was used to identify differentially expressed genes (DEGs). Results were considered significant if the Benjamini–Hochberg adjusted p-value, represented by false discovery rate, was <0.01 and fold change was >2. DEGs were used in the enrichment analysis and significance was detected using Fisher’s exact test. The cases were grouped by cancer type, and the differential gene expression characteristics were analyzed before and after SBRT. Then, we further analyzed the differential expression of each case before and after SBRT. Based on paired-end RNA-seq data, we aligned the fastq files that passed QC to the reference genome using the STAR software program and prepared a count matrix, which tallies the number of RNA-seq reads/fragments within each gene for each sample using StringTie and DESeq.

Fusion gene analysis was performed based on the RNA-seq data. The fusion point of each gene and the 5′- and 3′-chromosomal ends were examined. The count of read pairs supported the fusion including the multi-mapping reads and count of unique mapped reads on the fusion junction. Then, all reads were counted as maps on the fusion junction minus the polymerase chain reaction duplicated reads. In this study, fusion genes were detected using FusionCatcher. It is a tool used for finding novel and known somatic fusion genes in paired-end RNA-seq data in diseased samples collected from vertebrates that had available annotation data in the Ensembl database (12).

The T-cell receptor (TCR) comprises a heterodimer of two chains (αβ or γδ), both of which are products of variable (V), diversity (D), and joining (J) (V(D)J) recombination. This somatic rearrangement occurs only in the T-cell genome and produces an extremely diverse repertoire of TCRs. The most variable region in TCR is CDR3, which has a critical role in antigen recognition. In this study, we utilized a method to assemble *de novo* the CDR3 sequences generated from TCR locus transcripts in paired-end RNA-seq data by deep sequencing. This method first maps the reads to the human genome and searches for read pairs, with one mate properly mapped to a TCR gene and the other mate unmappable to the genome, potentially owing to the V(D)J recombination. It then initiates pairwise comparisons between the unmapped reads and constructs a read-overlap matrix, represented by an undirected graph, with each node corresponding to a read and edges indicating partial sequence overlap between the two connected reads. This graph is further divided into disjoint cliques to represent potentially different CDR3 sequences. Finally, all reads in each clique were assembled to obtain the contigs of DNA sequence and annotate them with information, including amino acid sequence and the associated V and J genes. TCR repertoire analysis was performed using iXCR (version 3.0.5) that processes large immune data from raw sequences into quantitated clonotypes (13).

Tumor-infiltrating immune cell analysis was performed using xCell (14), which is a gene signature-based method learned from thousands of pure cell types from various sources and applies a novel technique to reduce the associations between closely related cell types. Based on transcriptome sequencing data, a cell-type enrichment analysis of gene expression data for 64 immune cell types was performed using xCell.

### Exome Sequencing

WES assays of tumor biopsy samples and peripheral white blood cells were performed to analyze genomic mutations, copy-number alterations, and select fusions involving cancer-associated genes. The paired-end reads were mapped to the human reference genome GRCh37.75 using BWA (version 0.7.15-r1140) with default parameters. Variant calling was executed using GATK MuTect2 (v3.7-0-gcfedb67), Verdict (v1.4.8-0), and VarScan (v2.4.2). Variants were annotated with ANNOVAR (2016-02-01), according to the genomic coordinates GRCh37.75, and complex variants were further annotated with SnpEff (v4.3). Then, we examined whether the variants were present in the dbSNP (v147) common database. Variants not found in the dbSNP database were further filtered using the ClinVar (20181028) database, Cosmic (70), 1000g_EAS, ExAC_ALL, and ExAC_EAS. Pathogenic variants were annotated as “likely pathogenic,” “pathogenic,” or “drug response” in the ClinVar database. Raw exome sequencing data were also used to analyze the copy-number variation (CNV), which was performed using the CNVkit (v0.9.6).

Tumor mutational burden (TMB) assay was performed to examine the number of base substitutions, deletions, and insertion mutations in the protein coding region. The variations determined on NGS analysis include three parts: germline variation, somatic variation, and sequencing error. The original somatic variation set must be filtered before performing subsequent analysis. The filtration criteria are as follows:

1. The ratio is greater than 3% for mutations present in the Cosmic database and greater than 5% for mutations absent in the Cosmic database.
2. 1000g2015aug_all, ESP6500si, 1000g_EAS, ExAC_ALL, and ExAC_EAS mutation rate in the five normal human mutation databases is less than 1%.
3. Synonymous mutations must be removed from the dataset, and the mutation site is controlled only in the exonic and splicing regions.

When the above filtration criteria are met, it can be ensured that the mutations for subsequent analysis are rare in the normal population. The TMB value was obtained in accordance with the TMB algorithm from FMI^①^. The FMI method (FoundationOne CDx™) was approved by the Food and Drug Administration in 2017 for clinical drug selection decision making. Furthermore, raw exome sequencing data were also used to search for CNVs and single-nucleotide variants (SNVs)/Indel analysis.

### Immunohistochemical Analyses

The biopsy specimens were embedded in paraffin, and immunohistochemical (IHC) staining was performed on 5-μm-thick tissue sections using affinity mouse monoclonal antibodies against CD8 (clone C8-144B, dilution 1:1; Maixin Biotech, Fuzhou, China) and PD-L1 (clone 22c3, dilution 1:40, Agilent Tech, Inc., USA). Tissue sections were deparaffinized and stained using the Ventana BenchMark ULTRA in automatic mode (Roche Diagnostics GmbH, Germany) for analyzing CD8+ T-cell infiltration and PD-L1 expression in tumor cells.

### Statistical Analysis

The difference between the two groups was analyzed using Student’s t-test, and the comparisons were two-sided, with a significance level of 0.05. Pearson’s correlation coefficients between TMB and neoantigen, TCRseqs, and T cell were calculated as follows: γ<0.30=low, 0.3<γ<0.60 =moderate, and γ>0.60=high.

## Results

Two paired samples did not meet the QC for pathological examination because of not enough tumor cells. One paired sample for lung metastasis from rectal cancer could not be analyzed using WES and RNA-seq due to the bad quality or quantity of DNA/RNA. Ten paired samples were analyzed with WES, and RNA-seq was detected in nine paired samples except in case 2 (**Supplementary 1**). Clinical and pathologic characteristics of ten patients whose samples underwent WES and RNA-seq analysis are shown in **Table 1**.

**Table 1:** Clinical and pathologic characteristics of patients.

No.	Diagnosis	Clinical stage*^a^*	Histology	Lesion of irradiation	Fraction of radiotherapy	WES	RNA-seq
Case 1	EC	Metastasis	ASC	metastastic lesion	60 Gy/10F	Yes	No
Case 2	CC	Metastasis	SCC	Metastatic lesion	60 Gy/10F	Yes	Yes
Case 3	NSCLC	T4N3M1	AC	Primary lesion	60 Gy/10F	Yes	Yes
Case 4	CRC	Metastasis	AC	Metastatic lesion	60 Gy/10F	Yes	Yes
Case 5	NSCLC	T2N2M1c	AC	Primary lesion	60 Gy/10F	Yes	Yes
Case 6	CRC	TxNxM1b	AC	Metastatic lesion	60 Gy/10F	Yes	Yes
Case 7	NSCLC	T2N3M1c	AC	Primary lesion	60 Gy/10F	Yes	Yes
Case 8	CRC	Metastasis	AC	Metastatic lesion	60 Gy/10F	No	No
Case 9	NSCLC	T2N3M1b	AC	Primary lesion	60 Gy/10F	Yes	Yes
Case10	CRC	Metastasis	AC	Metastatic lesion	60 Gy/10F	Yes	Yes
Case11	NSCLC	T2N3M1c	AC	Primary lesion	60 Gy/10F	Yes	Yes

EC, Esophageal cancer; CC, Cervical cancer; NSCLC, non-small-cell lung cancer; CRC, colorectal cancer; ASC, Adenosquamous cancer; SCC, Squamous cell carcinoma; AC, Adenocarcinoma.

a Cancer stage were assigned in accordance with American Joint Committee on Cancer (AJCC) TNM Staging Classification for NSCLC, cervical cancer, colorectal cancer, and esophageal cancer (8th ed, 2017).

### SBRT Causing New Genomic Mutations but Not Increasing TMB

**Figure 1:**
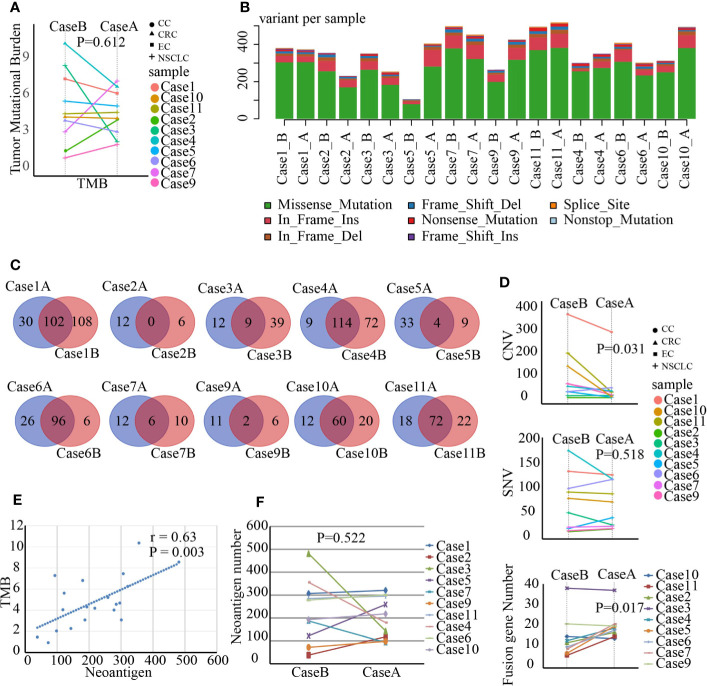
Gene mutations, fusion genes, and neoantigen prediction were analyzed by whole-exome sequencing and RNA-seq in paired tumor samples before and after stereotactic body radiotherapy (SBRT). *_B indicates the values before SBRT, *_A indicates the values after SBRT. **(A)** Changes in the tumor mutation burden (TMB) value after SBRT (*P*=0.612). **(B)** The number of variants in each sample is shown in a stacked bar plot and the variant types in a boxplot and summarized based on variant classification. **(C)** Number of unique mutations owned in paired tumor samples compared to the somatic mutations of peripheral white blood cells and number of unique mutations in Venn plots for each patient. The left number indicates unique mutations in tumor tissue after SBRT compared to those in the paired sample before radiation treatment, while the right number shows unique mutations in the tumor tissue before SBRT compared to the paired sample after receiving SBRT. The middle number specifies co-owned mutations in paired samples before and after SBRT. **(D)** Differences in copy-number variation (CNV; *P* = 0.031), single-nucleotide variant (SNV; *P* = 0.518), and fusion gene (*P* = 0.017) in the tumor microenvironment (TME) before and after SBRT. **(E)** Linear relationship between TMB and neoantigen and the value of neoantigen prediction (r = 0.63, *P* = 0.003). **(F)** Comparison of neoantigen number before and after radiotherapy (*P* = 0.522).

Radiation can trigger new mutations. Whether SBRT could improve TMB remains unclear. WES as a standard method for TMB analysis can detect somatic mutations present within the entire exome. We analyzed a series of genomic variations including CNVs, SNVs, and fusion genes using WES in tumor tissue before and after SBRT. Surprisingly, the TMB value of the tumor tissue did not increase coincidentally in 10 samples treated with SBRT (**Figure 1A**, *P*=0.612). However, all 10 tumor samples showed new mutations after radiation (**Figures 1B, C**). Then, we analyzed the mutations or aberrations of the genome in detail. The results (**Figure 1D**) showed that SNV in tumor deposits had no significant difference before and after irradiation (*P*=0.518); the CNV of tumor specimens that received SBRT decreased significantly (*P*=0.031). In contrast, the fusion genes increased significantly in tumor samples after receiving radiation (*P*=0.017). The absence of significant changes in TMB may be due to inconsistent changes in CNVs and fusion genes. The changes in neoantigen number was similar to TMB in paired samples (**Figure 1E**, r=0.63, *P*=0.003). Moreover, the neoantigen number in paired samples had no obvious changes (**Figure 1F**, *P*=0.522). These results implied that SBRT can trigger only new mutations and induce neoantigen rather than increasing the TMB in the TME.

### Increased PD-L1 Expression With No Significant Change in CXCL10, CXCL16, MHC-I, IFNs, and IFNs Receptors in the TME After SBRT

**Figure 2:**
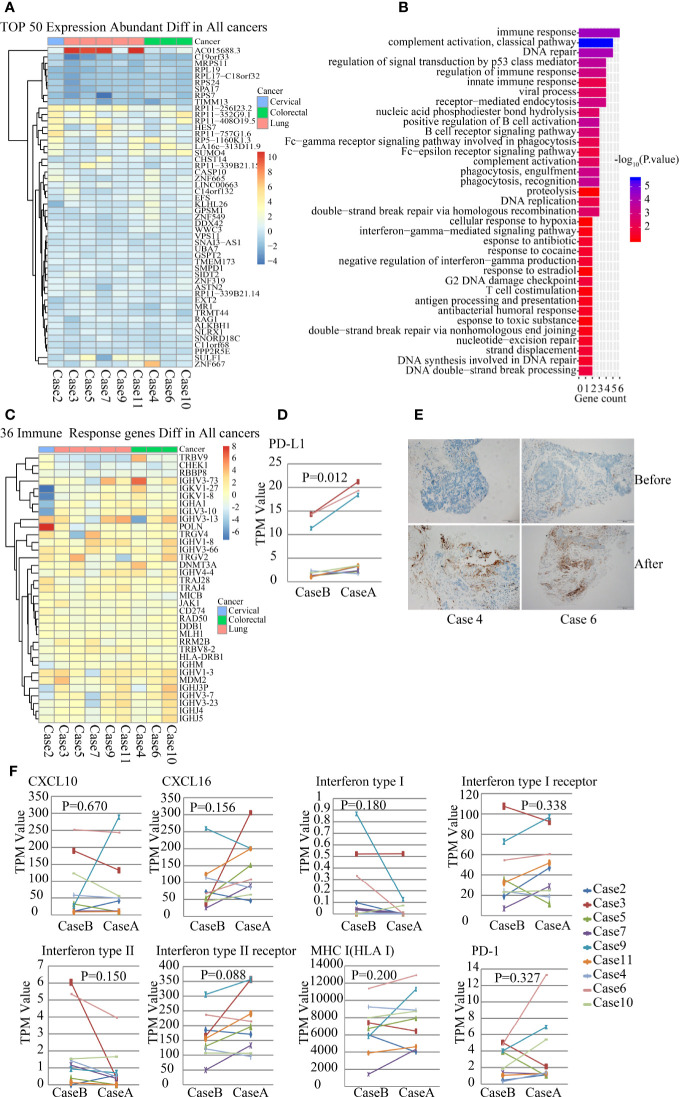
Gene expression analysis of the paired samples before and after stereotactic body radiotherapy (SBRT) by RNA-seq. *_B indicates the values before SBRT; *_A indicates the values after SBRT. **(A)** Heatmap of 50 differentially expressed genes abundant in nine paired tumor samples. **(B)** Gene ontology (GO) enrichment pathway analysis of nine paired tumor samples. **(C)** Heatmap of the expression of 36 immune responsive genes abundant in nine tumor samples before and after SBRT. **(D)** Differences in PD-L1 expression in the tumor microenvironment (TME) before and after SBRT (P = 0.012). **(E)** PD-L1 expression was detected in tumor tissue using IHC and it increased significantly in tumor cells after SBRT compared to before SBRT. **(F)** Comparison of the expression of CXCL10, CXCL16, IFN I receptor, IFN II receptor, IFN I, IFN II, MHC-1, and PD-1 in the TME before and after SBRT.

In addition to WES analysis, whole transcriptomes in TME were detected with RNA-seq. We analyzed the differential expression characteristics of the tumor tissue before and after radiation treatment, and DEGs were analyzed using paired-sample t-test. The top 50 DEGs are listed in the heat map (**Figure 2A**). Then, we performed gene ontology (GO) enrichment analysis of these altered genes by biological process classification. The most enriched term was the immune response gene (six genes, *P*=4.67E-05), followed by gene of DNA repair (five genes, *P*=2.55E-06) and regulating gene of signal transduction by p53 class mediator (five genes, *P*=7.71E-05) (**Figure 2B**). The differential genes of immune responses included many TCRs and immunoglobulin superfamily as listed in **Figure 2C**. Moreover, PD-L1 (CD274) expression in the TME significantly increased after SBRT than before radiation (**Figure 2D**, *P*=0.012). To confirm this result, we performed an IHC assay and found that PD-L1expression in tumor tissue increased significantly after SBRT (**Figure 2E**).

Furthermore, several studies have revealed that chemokines and pro-inflammatory factors such as CXCL10, CXCL16, IFNs, and its receptors could be induced by radiation in mice models and were essential for recruiting and activating CD8+ T cells (15, 16). However, in this study, expression of IFN-I, IFN-II, CXCL10, CXCL16, IFN-I receptor, and IFN-II receptor did not show significant differences in paired samples before and after SBRT (**Figure 2F**). MHC-I expression in tumor tissues that received SBRT also showed no significant differences when compared to that before radiation (**Figure 2F**), although it can be enhanced by radiation, thus inducing antitumor immunotherapy in murine tumor transplant models (17). Furthermore, there was no significant difference in PD1 expression in the TME before and after SBRT (**Figure 2F**).

### Effect of SBRT on the Diversity of TCR Repertoire and Frequency of TCR in the TME

**Figure 3:**
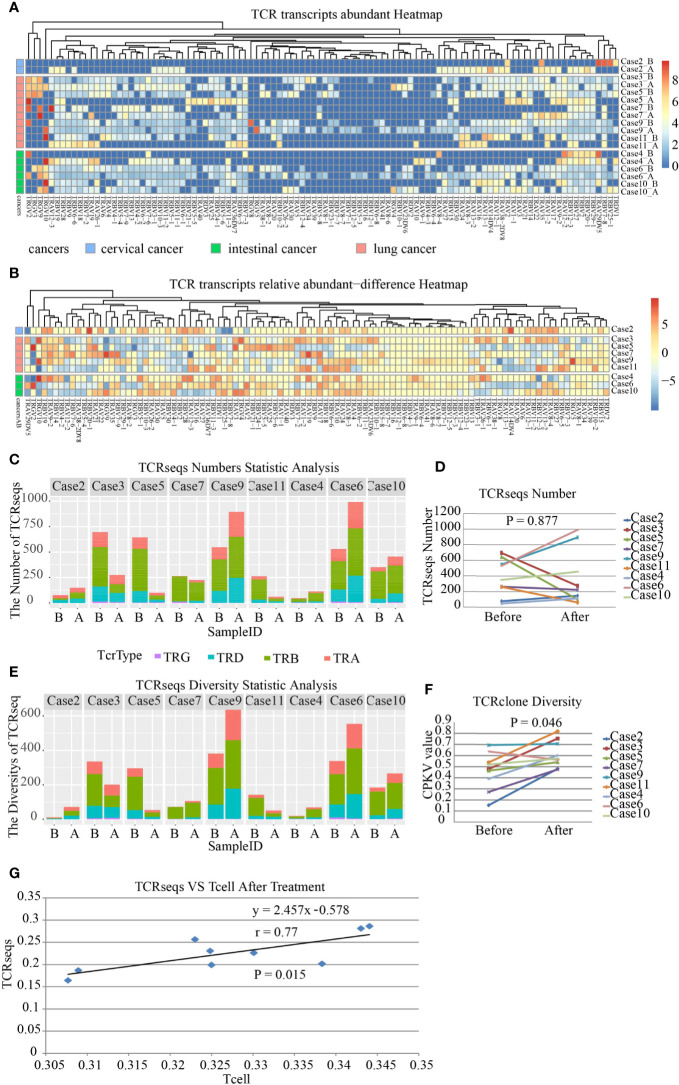
Transcript abundance and repertoire diversity of T-cell receptor (TCR) in nine patients before and after stereotactic body radiotherapy (SBRT). TRA, TRB, TRD, and TRG mean Vα, Vβ, Vγ, and Vδ segment usage of TCR, respectively. *A represents samples after SBRT, while *B represents samples before SBRT. **(A)** Frequency and distribution of TRA, TRB, TRD, and TRG gene usage and productive sequences in all samples. **(B)** The heatmap shows the difference in abundance in TRA, TRB, TRD, and TRG transcripts in each case after SBRT compared to that before radiation. **(C)** Transcript abundance of TCRs comprising TRA, TRB, TRD, and TRG in each sample on the x axis; the value on the y axis represents the number of TCRseqs. **(D)** Comparison of TCR transcript abundance in the tumor microenvironment (TME) before and after SBRT (*P* = 0.877). **(E)** Diversity of TCR repertoire in each sample. **(F)** Diversity of TCR repertoire assessed by CPKV value after SBRT increased significantly (*P* = 0.046). The values on the y axis represent the diversity of TCRseqs. **(G)** Relationship between the amount of TCRseqs and the relative amount of T cell (R^2^ = 0.594). Each point represents one sample. The value on the x axis represents the amount of TCR clones, while the value on the y axis represents the amount of TCRseqs.

The landscape of neoantigens is thought to determine the immunogenicity of cancers, and in particular, the antitumor responses mediated by T-cells. Therefore, we characterized the TCR repertoire in the TME. The diversity and frequency of TCRs, including TRA, TRB, TRD, and TRG, reflect the diversity and activity of cellular immunity. We amplified and sequenced the TCR repertoire including approximately 260 distinct TCR genes in paired samples. The results showed that TCR gene expression in the TME was modified by SBRT (**Figures 3A, B**). However, no significant difference was observed in the frequency of TCRs in tumor tissues after receiving SBRT when compared with that before irradiation (**Figures 3C, D**, *P*=0.877), although the frequency of TCRs in the tumor samples increased in five cases that underwent SBRT.

The diversity of TCR repertoire was analyzed using the ratio of the number of unique TCRseqs to the number of total TCRseqs as an indicator. The indicator was defined as the CPKV value and was more accurate than the CPK value (18). The results demonstrated that the diversity of TCR repertoire in samples treated with SBRT was significantly higher than that before irradiation (**Figures 3E, F**, *P*=0.046). This finding indicated that SBRT might promote T-cell-mediated immune responses against tumors by increasing the diversity of TCR repertoire in the TME. Lastly, we analyzed the consistency between TCRseqs and T cell and found a high consistency between them (**Figure 3G**, r=0.77, *P*=0.015).

### Profiling Tumor-Infiltrating Immune Cells in the TME Before and After SBRT

**Figure 4:**
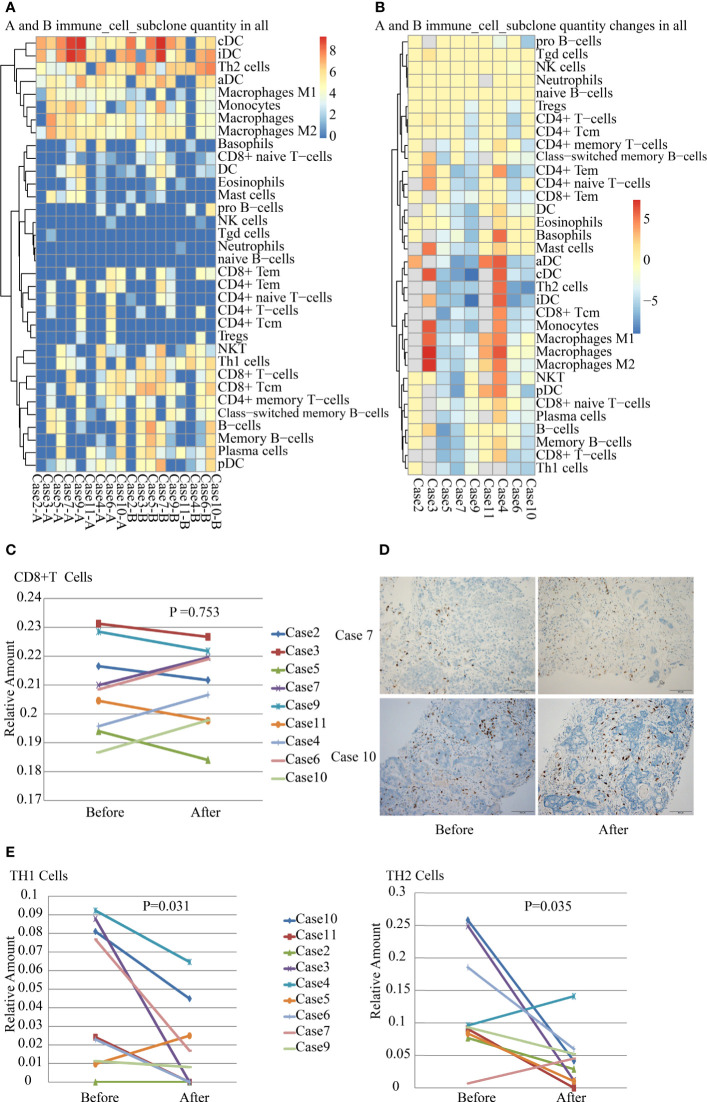
Analysis of tumor-infiltrating immune cells in nine paired samples before and after stereotactic body radiotherapy (SBRT). **(A)** Inferred composition of 34 immune cell subsets in samples of each patient before and after SBRT. The results were generated from gene expression data for 64 immune cell types using xCell. **(B)** Differences in the number of tumor-infiltrating immune cell subsets before and after SBRT in nine patients. **(C)** Differences in CD8+ T-cell infiltration in the tumor microenvironment (TME) before and after SBRT (*P* = 0.753). **(D)** CD8+ T cells infiltration were detected by IHC in tumor tissues before and after SBRT. **(E)** Differences in TH1 cell (P = 0.031) and TH2 cell (P = 0.035) infiltration in the TME before and after SBRT.

Tumor-infiltrating immune cells are associated with promoting or inhibiting antitumor immune response, which corresponds to good or poor outcomes in different cancers (19). We analyzed tumor-infiltrating immune cells including 34 subsets in paired tumor samples. The results revealed striking differences in the immune cells in the TME in different patients after SBRT (**Figures 4A, B**). There were no significant changes in the number of tumor-infiltrating NK and Treg cells in most paired samples before and after irradiation. The number of tumor-infiltrating CD8+ T cells in the TME did not increase remarkably a week after SBRT (*P*=0.753). On the contrary, it reduced in five samples after radiotherapy (**Figure 4C**, *P*=0.753). The result was confirmed by the IHC detection of CD8+ T cells in TME (**Figure 4D**). In addition, only a significant reduction in TH1 and TH2 cells was observed in tumor deposits receiving SBRT (**Figure 4E**). The changes in tumor-infiltrating immune cell number implied that the responses of immune cell subsets vary in different patients after SBRT.

## Discussion

The success of ICIs combined with radiotherapy in several types of cancers ignited the interest of oncologists to explore the effects of immunotherapy fueled by radiotherapy. This phenomenon has also been verified indirectly in mouse tumor models (20, 21). Thus, understanding the immunologic effects induced by radiation is important to design rational combination strategies of radiotherapy and immunotherapy.

Irradiation may increase nonsynonymous mutation burden and trigger neoantigen production in cancer cells, possibly favoring *in situ* vaccine development and TME reprogramming (22). Rizvi et al. found that a higher TMB in tumors was associated with an improved pembrolizumab effect in NSCLC patients (23). Therefore, we detected the genomic variance of tumor tissues a week after SBRT. Beyond our conjecture, SBRT did not promote TMB increase in the TME, although the CNV decreased significantly and the fusion genes increased markedly after radiation. The changes in TMB, CNV, and fusion genes could be due to double-strand breaks (DSBs) of DNA induced by SBRT in cancer cells, leading to the repair of DNA DSBs. While the radiation-induced production of DNA damage is linear, the radiobiological effects are generally non-linear. Cancer cells repair DNA damage and survive by inducing DNA repair and cell cycle arrest or start cellular death without DNA repairing after irradiation treatment (24). New nonsynonymous gene mutations were observed in each patient. Thus, SBRT may not improve the quantity of TMB and neoantigen in the TME but trigger new nonsynonymous mutations, possibly inducing tumor-specific neoantigen development.

Biological responses of cancer cells to radiation involved damage, repair, apoptosis, or necrosis, leading to the activation or inhibition of signal transductions of DNA repair, metabolism, and cell cycle arrest (25). In this study, RNA-seq results demonstrated that several genes regulating cancer proliferation, invasion, and immune response might be under-expressed or over-expressed in tumor tissues after SBRT. Expression of a large number of immune-related genes has changed. Tumor immune microenvironment comprises inflammatory factors, infiltrating immune cells, and stroma cells and can be reprogrammed by radiotherapy (26). Expression of CXCL10, CXCL16, IFNs, and IFN receptors could be induced by irradiation in an experimental animal model (15, 16, 27, 28). However, expression of CXCL10, CXCL16, IFNs, and IFN receptor showed various changes, including an increase and decrease in the TME after SBRT. There was no significant difference in the expression of CXCL10, CXCL16, IFNs, and IFN receptors before and after SBRT. This result was contrary to that of other experimental animal studies but was similar in relation to that of tumor-infiltrating immune cells in this study.

Tumor-infiltrating lymphocytes (TILs) are integral components of the TME and correlate with response to immune therapy (29, 30). Neoadjuvant chemoradiation therapy increased the density of TILs in post-treatment resected rectal cancer specimens (31). However, our results showed that SBRT did not promote CD8+ T-cell and NK-cell infiltration in the TME. With regard to infiltrating immune cells in the TME in this study, which were different from that reported in other studies, the discrepancy might be due to the differences in the fractionated dosage of radiotherapy or time point of sample collection.

The antitumor responses of effector T-cells comprised recognizing tumor antigen and attacking cancer cells, which are associated with MHC-I, PD-1/PD-L1 axis, and TCR. The increase in MHC-I expression in tumor tissues after SBRT promoted recognition of *in situ* tumor-specific antigen to CD8+ T cells. It was supported by the improvement in the diversity of TCR repertoire in the TME after SBRT. The high diversity of TCR repertoire reflected a better immune status, which indicated clinical benefit in cancer patients (32). Based on the result of MHC-I and TCR repertoire diversity, this study supported that SBRT promoted the antitumor immune response. An important study finding was the upregulation of PD-L1/PD-1 expression in the TME, which was supported by the results of Sato’s research that revealed that DNA DSBs upregulated PD-L1 expression in an ATM/ATR/Chk1-dependent manner (33).

This is the first study to report the direct effect of SBRT on genomic mutation, differences in gene expression, and tumor immune microenvironment. Notably, the diversity of TCR repertoire increased and PD-L1 expression upregulated in the TME after SBRT. SBRT induced neo-mutation and new fusion gene in cancer cells, which induced the generation of tumor-specific neoantigen but did not increase TMB in tumor tissues. It displayed complex changes in tumor-infiltrating immune cells and expression of immune-regulating factor in the TME after receiving SBRT. However, SBRT did not increase CD8+ T-cell infiltration and IFN-I/II expression in tumor tissues a week after radiation. Certainly, this study had some limitations. The study used a small sample size and did not perform a dynamic observation of the TME after SBRT. A few metastatic tumors to lung was grouped in analysis of this study. The changes of genome and tumor immune microenvironment induced by SBRT could not be considered as a specific results of SBRT because it lacked of a control group for conventional radiotherapy. Hence, further studies are warranted to address these concerns.

## Data Availability Statement

The data presented in the study are deposited in the NCBI GEO database repository, accession number: GSE162946. (https://www.ncbi.nlm.nih.gov/geo/query/acc.cgi?acc=GSE162946).

## Ethics Statement

The studies involving human participants were reviewed and approved by the Ethics Committee of Xinqiao Hospital (Ethical No: 2019-Res 032-1), Chongqing 40037, China. Written informed consent for participation was not required for this study in accordance with the national legislation and the institutional requirements.

## Author Contributions

PZ and DC performed the collection and preservation of samples including analysis of some parts of the results. BZ took part in the analysis and interpretation of the TCR results. WC wrote the methods of RNA-seq and WES. QX analyzed and interpreted the tumor-infiltrating immune cell results. YW and QT performed the pathological diagnosis for the patients. BY, XZ, CH, and HZ provided treatment to patients. GL conceived and designed the experiments, summarized the results, guided SBRT for the patients in this study. He also wrote and revised this manuscript. All authors contributed to the article and approved the submitted version.

## Funding

This work was supported by the Medical Research Foundation Project of CQ Health Commission (No. 2019ZDXM043) and Science and Technology Innovation Ability Promotion Project of AMU (No. 2019XLC2011).

## Conflict of Interest

Author WC was employed in Zhongyuan Union Clinical Laboratory Co. Ltd.

The remaining authors declare that the research was conducted in the absence of any commercial or financial relationships that could be construed as a potential conflict of interest.
