# Supplementary material for: Stereotactic Body Radiotherapy Is Effective in Modifying the Tumor Genome and Tumor Immune Microenvironment in Non-Small Cell Lung Cancer or Lung Metastatic Carcinoma
Source: Front Immunol. 2021 Jan 22;11:594212. doi: 10.3389/fimmu.2020.594212 (PMC7862546; doi:10.3389/fimmu.2020.594212)
Supplement: Supplementary file 1 [file Table_1.doc]

**Supplementary 1: Quality and quantity of extracted DNA and RNA from samples**

**Table S1: Quantity of extracted DNA from samples**

| **No.** | **DNA concentration (ng/μl)** | **DNA volume（μl）** | **DNA quantity(ng)** |
| --- | --- | --- | --- |
| case 1 B | 18.3 | 120 | 2196 |
| case 2 B | 7.44 | 120 | 892.8 |
| case 3 B | 38.4 | 120 | 4608 |
| case 4 B | 5.42 | 120 | 650.4 |
| case 5 B | 6.06 | 120 | 727.2 |
| case 6 B | 12.5 | 120 | 1500 |
| case 7 B | 10.5 | 120 | 1260 |
| case 8 B | 9.36 | 120 | 1123.2 |
| case 9 B | 5.16 | 120 | 619.2 |
| case 10 B | 17.3 | 120 | 2076 |
| case 11 B | 18 | 120 | 2160 |
| case 1 A | 20.8 | 120 | 2496 |
| case 2 A | 5.16 | 120 | 619.2 |
| case 3 A | 14.3 | 120 | 1716 |
| case 4 A | 5.52 | 120 | 662.4 |
| case 5 A | 8.14 | 120 | 976.8 |
| case 6 A | 12.7 | 120 | 1524 |
| case 7 A | 6.12 | 120 | 734.4 |
| case 8 A | 2.98 | 120 | 357.6 |
| case 9 A | 4.04 | 120 | 484.8 |
| case 10 A | 12 | 120 | 1440 |
| case 11 A | 18.2 | 120 | 2184 |

Case * B: case * before SBRT, Case * A: case * after SBRT

Table s2: Quality and quantity of extracted RNA from samples

| No. | RNA concentration (ng/ul) | RNA volume (ul) | RNA quantity (ug) | RNA OD260/280 | RNA OD260/230 | RIN | Conclusion |
| --- | --- | --- | --- | --- | --- | --- | --- |
| case 1 B | 146 | 14 | 2.044 | 1.96 | 1.83 | 8.2 | A |
| case 2 B | 48 | 14 | 0.672 | 1.5 | 1 | 6.4 | A |
| case 3 B | 172 | 14 | 2.408 | 1.86 | 1.49 | 7.1 | A |
| case 4 B | 32 | 14 | 0.454 | 1.9 | 1.2 | 6.5 | A |
| case 5 B | 153 | 14 | 2.136 | 1.69 | 1.44 | 6.8 | A |
| case 6 B | 72 | 14 | 1.008 | 1.64 | 0.89 | 8.6 | A |
| case 7 B | 33 | 14 | 0.46 | 2 | 1.36 | 7.2 | A |
| case 8 B | 62 | 14 | 0.868 | 1.57 | 0.65 | 1.1 | D |
| case 9 B | 74 | 14 | 1.036 | 1.96 | 1.23 | 8.3 | A |
| case 10 B | 125 | 14 | 1.75 | 1.7 | 1.13 | 7.1 | A |
| case 11 B | 284 | 14 | 3.976 | 1.82 | 1.69 | 7.7 | A |
| case 1 A | 52 | 14 | 0.728 | 0.86 | 0.5 | 0 | D |
| case 2 A | 50 | 16 | 0.8 | 2.21 | 1 | 7.9 | A |
| case 3 A | 89 | 14 | 1.242 | 1.77 | 1.5 | 8.5 | A |
| case 4 A | 51 | 14 | 0.72 | 1.56 | 1.14 | 7.4 | A |
| case 5 A | 76 | 14 | 1.064 | 2 | 1.65 | 6.3 | A |
| case 6 A | 63 | 14 | 0.882 | 1.86 | 1 | 8.5 | A |
| case 7 A | 61 | 14 | 0.856 | 1.67 | 0.83 | 6.7 | C |
| case 8 A | 52 | 14 | 0.74 | 1.42 | 0.81 | 2.6 | D |
| case 9 A | 194 | 14 | 2.716 | 1.8 | 1.83 | 8.6 | A |
| case 10 A | 188 | 14 | 2.632 | 1.88 | 1.34 | 8.4 | A |
| case 11 A | 116 | 14 | 1.624 | 1.77 | 1.35 | 7 | A |

Case * B: case * before SBRT, Case * A: case * after SBRT, A: accept, D: defect

Red indicates that the sample does not meet the test quality and will be removed in the next analysis.
